# Are there atypical sites of IgG4 related disease in head and neck region? Personal experience and literature review

**DOI:** 10.1007/s00405-024-09188-6

**Published:** 2025-01-11

**Authors:** Melania Bertolini, Francesco Buono, Alice Galli, Diego Bagnasco, Luca Guastini, Monica Feltri, Frank Rikki Mauritz Canevari

**Affiliations:** 1 Department of Otolaryngology and Head and Neck Surgery, IRCSS AOU San Martino, University of Genoa, Largo Rosanna Benzi 10, 16132 Genoa, Italy; 2 Department of Internal Medicine, Allergy & Respiratory Diseases, IRCCS AOU San Martino-IST, University of Genoa, Largo Rosanna Benzi 10, 16132 Genoa, Italy; 3 Unit of Pathology, IRCCS AOU San Martino-IST, University of Genoa, Largo Rosanna Benzi 10, 16132 Genoa, Italy

**Keywords:** Immunoglobulin G4-related disease (IgG4-RD), Head and neck, Atypical sites, Clinicopathological manifestations

## Abstract

**Purpose:**

Immunoglobulin G4-related disease (IgG4-RD) is a complex systemic fibroinflammatory condition with different clinical manifestations affecting multiple organ systems. Despite its rarity, the disease presents diagnostic and therapeutic challenges due to its mimicry of malignancies and other immune-mediated disorders. The 2019 American College of Rheumatology/European League Against Rheumatism Classification Criteria for IgG4-Related Disease is the current state of art to confirm the diagnosis of IgG4-RD even in the absence of histological analysis. However, this classification excludes atypical sites, focusing on the more typical ones, even in case of histological confirmation. In the ENT field, several localizations of this disease have been described.

**Methods:**

We report two clinical cases at the Otolaryngology Unit of IRCCS San Martino Hospital, Genoa affected by IgG4-RD arising in atypical sides of the head and neck region. Additionally, we perform a clinical review of the current literature.

**Discussion:**

The review provides an extensive overview of IgG4-RD, encompassing epidemiology, clinical manifestations, diagnostic approaches, and therapeutic strategies. We discuss the evolution of diagnostic criteria, emphasizing the necessity of interdisciplinary collaboration among clinicians, radiologists, and pathologists for accurate diagnosis. Diagnostic imaging plays a crucial role, with characteristic radiological patterns aiding in the identification of affected organs. However, definitive diagnosis often requires histopathological confirmation, highlighting the importance of biopsy in challenging cases. We also focus on the treatment of IgG4-RD which poses significant challenges, with glucocorticoids remaining the cornerstone of therapy. Emerging steroid-sparing agents such as rituximab and Dupilumab, show promising results in refractory or recurrent disease.

**Conclusions:**

IgG4-RD is a multisystemic fibroinflammatory disease that can potentially affect any part of the body. The 2019 ACR/EULAR 3-stage classification criteria for IgG4-RD considers only a few head and neck sites. Therefore, it is of paramount importance that neurosurgeons, head and neck surgeons, and oral and maxillofacial pathologists are familiar with the clinicopathological manifestations of IgG4-RD in these sites to avoid misdiagnosis and inappropriate treatment, which can lead to a decrease in patients’ quality of life. To our knowledge, there are no risk factors or genetic predispositions. Further studies are needed to elucidate the pathophysiology of IgG4-RD with the aim of providing a targeted therapy that could spare steroid-related effects and reduce relapses.

## Introduction

Immunoglobulin G4-related disease (IgG4-RD) is an uncommon systemic, immune-mediated fibroinflammatory disease that can involve almost any anatomic site. Due its multiorgan manifestation, it can be misdiagnosed as malignancy, infection, or other immune-mediated condition such as Sjogren’s syndrome or ANCAs associated vasculitis [1]. Indeed, many practitioners are often involved in the evaluation of this condition.

First recognized as a distinct disease in 2003 [2], the subsequent decade has seen an increase in knowledge about its clinicopathologic features.

Epidemiology is still not well understood; however reported data indicate a male predilection after the fifth decade of life. A large epidemiologic study from Japan demonstrated a lower male to female ratio of 1:0.77 and an average age of onset of 58.8 years noting that only 10% of cases presented before the age of 40. Incidence and prevalence data still lack in detail, although Uchida et al. estimate the incidence in Japan to be approximately 0.28–1.08/100,000 population with 336–1300 newly diagnosed patients per year [3].

According to the site of involvement, it can present with various symptoms that overlap with other conditions and can lead to organ dysfunction, organ failure, and death. Traditionally it presents with a subacute manifestation with elevation of IgG4 serum level. In superficial sites, it can manifest as a visible mass, whereas in internal organs, it can increase its dimension and lead to dysfunction due to compression effect. Rarely can occur weakness and weight loss. Fever and signs of systemic illness are uncommon.

Therefore, the initial suspicion of IgG4-RD often relies on the correlation between an unexplained enlargement or swelling of one or several organs with laboratory abnormalities.

However, it is known that serum IgG4 can be normal in a good percentage of patients with clinical-pathological diagnoses of IgG4-RD, especially when certain organ systems and anatomic regions (e.g., the retroperitoneum) are involved [4]. On the other hand, other cases are early diagnosed with impressive level of IgG4 concentrations in blood tests. Given this heterogeneity, high IgG4 serum levels are no longer considered essential criteria for diagnosis.

Some radiologic findings can be strongly suggestive. Radiologic imaging shows a nonspecific fibroinflammatory process. Thought initially, imaging is not reliable in distinguishing IgG4-RD from other inflammatory or neoplastic processes, nowadays imaging patterns in specific body regions have been recognized to guide the correct diagnosis.

Obviously, radiologic features without clinical, serologic, or pathologic data are insufficient to offer a diagnosis.

To confirm the diagnosis, histopathological assessment is needed.

Characteristic pathologic features are storiform fibrosis, obliterative phlebitis, a lymphoplasmacytic infiltrate with prominent IgG4 + plasma cells, and an increased IgG4 + /IgG + plasma cell ratio [5]. Fine needle aspiration with cytologic analysis cannot confirm the diagnosis seen that the characteristic features are assessed only by histological analysis by core biopsy or open biopsy.

However, a standalone diagnosis is difficult and needs the support of other physicians.

To overcome possible confounding diagnosis, in 2019, the American College of Rheumatology/European League Against Rheumatism (ACR/EULAR) developed 3-step classification criteria for IgG4-RD [6] that demonstrated a specificity of 97.8% (95% CI 93.7–99.2%) and a sensitivity of 82.0% (95% CI 77.0–86.1%). The first step is based on inclusion criteria regarding clinical and radiological involvement of one of the ten typical organs. The second one regards exclusion criteria, that consist into clinical, serologic, biochemical, radiologic, and pathologic findings uncommon for this disease or rather the presence of another well documented disease. The third step, which is reached only if the inclusion and none of the exclusion criteria are met, involves eight weighted inclusion criteria domains addressing clinical findings, serologic results, radiology assessments, and pathology interpretations. A final threshold of 20 points shows high specificity and sensibility for IgG4-RD. Because of its completeness and complexity, one of the strengths of this classification is the possibility to give a reliable diagnosis without performing a biopsy.

However, a biopsy is still required in a significant number of cases to exclude mimickers, as well as in some IgG4-RD patients who do not fulfill the current classification criteria, especially when the disease affects less commonly involved organs. (Indeed, we reiterate that IgG4-RD is a multi-organ disease that can potentially involve any organ, although some sites are more typical.)

In support of this, we present two cases of patients who were admitted to our hospital center with IgG4-related disease in atypical head and neck locations. Furthermore, we provide an overview of the current literature, focusing on the typical radiological pattern, and separately describe the atypical head and neck localizations, emphasizing the importance of considering the possible diagnosis of IgG4-RD in these sites, despite the current diagnostic criteria. Finally, we also provide an overview of the etiopathogenesis, prognosis, and treatment, focusing on new emerging strategies.

## Materials and method

A literature review was performed using PubMed, Cochrane Library, Google Scholar and Scopus database and references cited in relevant articles. After a query for “ <IgG4-related AND (head or neck)> ” was entered, the search returned a total of 189 articles published in years 2011–2024. We included cases with at least one manifestation of the disease in the head and neck region at any site, therefore we excluded any IgG4-RD sites limited to other sites that head and neck region. There were no limitations regarding the study design seen the rarity of the disorder. We included systemic reviews, meta-analyses, retrospective studies and case series in the analysis.

## Case presentation 1

**Fig. 1:**
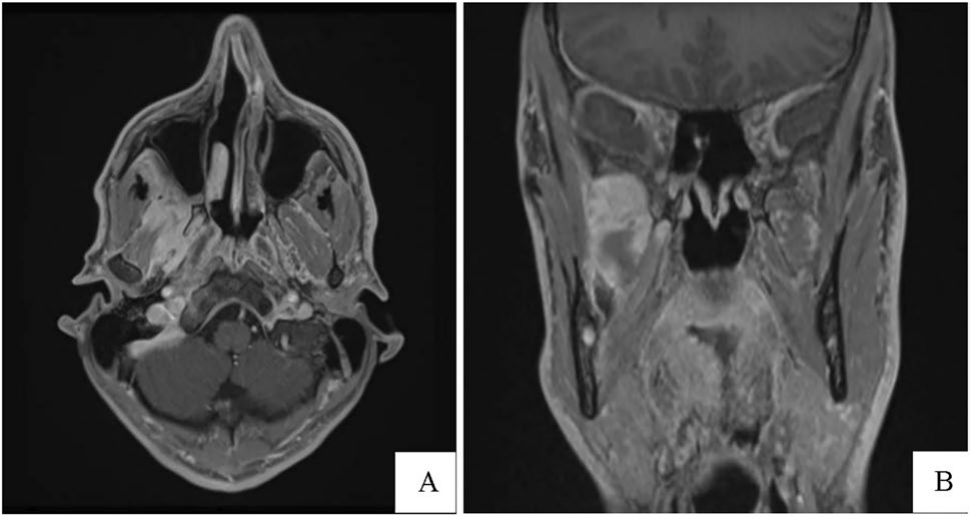
MRI axial (**A**) and coronal plane (**B**) showed T1W1 contrast enhancement uptake in the pterygopalatine fossa, extending medially to the sphenopalatine foramen and laterally to the infratemporal and masticatory space

A 33-year-old man was hospitalized for orbital cellulitis associated with exophthalmos. The patient complained of decreased visual acuity, headache, and right otalgia. ENT clinical examination was negative. Orbital magnetic resonance imaging (MRI) revealed contrast enhancement uptake in the pterygopalatine fossa, extending to the infratemporal and masticatory space. The pathological tissue reached the inferior orbital fissure and extended to the orbital apex and cavernous sinus. The infraorbital nerve canal, optic nerve, and vidian canal appeared to be involved (Fig. 1).

The initial hypotheses were adenoid cystic carcinoma (ACC) and lymphoma. However, PET-CT did not show significant accumulation in the right pterygopalatine fossa.

**Fig. 2:**
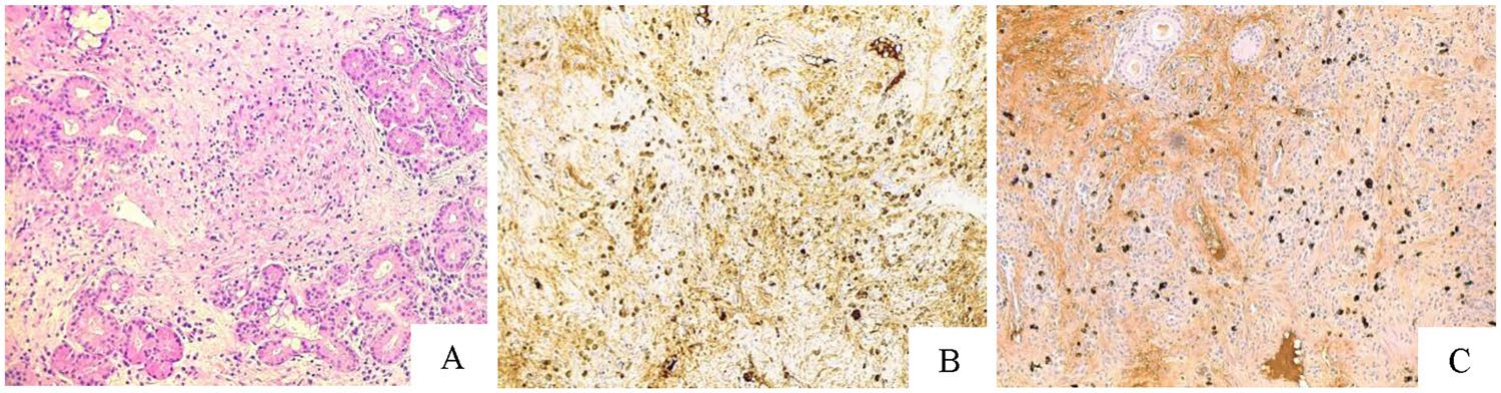
Hematoxylin–eosin-stained histological section shows lymphocytes and plasma cells infiltrating thickened and fibrotic vessels’ walls, with lumens’ obliteration (obliterative phlebitis, **A**). Immunohistochemistry stains with antibodies respectively against IgG (**B**) and IgG4 (**C**) allow to appreciate an IgG4/IgG positive plasma cell ratio of about 50%

He underwent surgery via a trans-ethmoidal-pterygoid-sphenoidal endoscopic approach to obtain a biopsy sample. The biopsy revealed an IgG/IgG4 ratio estimated to be about 50%, with varying numbers of IgG4 + plasma cells (up to 67 per high-powered field). The microscopic examination showed an inflammatory process with fibrosis, signs of obliterative vasculopathy, lympho-plasmocytic infiltrates, with an IgG4//IgG ratio in about 50% and without evidence of a neoplastic process: IgG4-related disease (IgG4-RD) was suggested (Fig. 2).

Histological criteria for IgG4-RD were met. However, according to the 3 Classification Criteria for IgG4-RD by ACR/EULAR and despite meeting all the other criteria, the diagnosis would be excluded for the location, which is deliberately not considered, as it is deemed atypical. Currently, the patient is undergoing high-dose steroid therapy along with rituximab, a monoclonal antibody targeting CD20.

## Case presentation 2

**Fig. 3:**
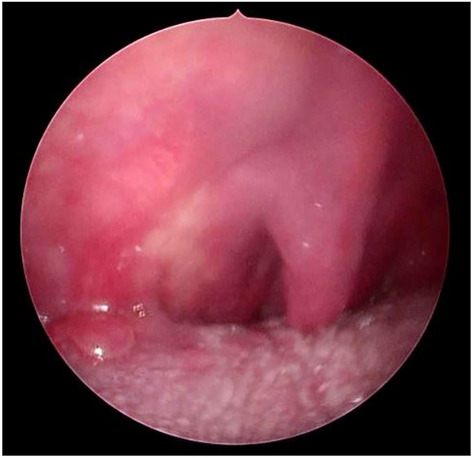
Oral endoscopy shows submucosal bulging at the right tonsillar pillar, the entire soft palate is involved, and the motility of the palatine velum is altered

A 59-year-old man complained of nasal obstruction associated with rhinolalia and oronasal reflux for three months. An ENT clinical examination revealed a submucosal bulge at the level of the right tonsillar pillar, with involvement of the entire soft palate and reduced motility of the palatine velum (Fig. 3).

Maxillary sinus CT and neck CT scans showed pathological tissue with diffuse contrast enhancement uptake of the soft palate and tonsil pillars bilaterally, extending posteriorly to the posterior wall of the nasopharynx. Maxillofacial MRI confirmed the presence of pathological tissue in the oropharynx and nasopharynx. An incisional biopsy of the soft palate under general anesthesia was performed. Histological findings showed an elevated number of IgG4 + plasma cells (98/HPF). At the level of the soft palate, an inflammatory infiltrate consisting of mature plasma cells and giant cellular epithelioid granulomas was described. Inside the granulomas, the presence of fungal hyphae recognizable with PAS and Grocott stains was reported.

**Fig. 4:**
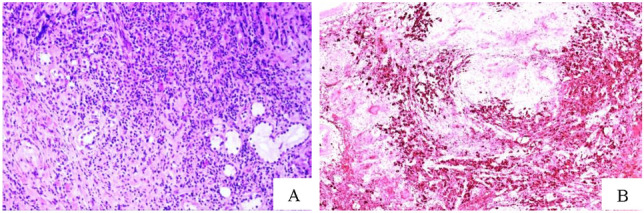
On hematoxylin–eosin e conspicuous plasma cell infiltrate was seen (**A**). IgG4 positive plasma cells were very numerous (98/HPF) and constituted more than 80% of the total amount (**B**)

Given the histological findings, the patient was referred for immunological evaluation and therapy (Rituximab). One year later, the patient returned to our ENT center due to the appearance of fibrous-purulent material at the level of the posterior wall of the nasopharynx, for which a new radiological examination was recommended. The new MRI showed substantial stability of the clinical features at the most cranial levels but worsening at the oral and hypopharyngeal levels, with involvement of the right hemi-tongue. Subsequently, due to the worsening of symptoms (dysphagia for solids and liquids), the patient was hospitalized and underwent PEG placement and a biopsy at the hypopharynx-larynx level. The histological result showed IgG4 + plasma cells (CD138 + , IgG + , and IgG4 +) > 100/HPF and an IgG4/IgG ratio > 40% (Fig. 4).

The patient also underwent a new CT scan, which revealed disease at the lung level. Given the suspicion of relapse and the poor improvement of symptoms, the dosage of Prednisone was increased to 50 mg/day, and a new cycle of Rituximab was started, and still ongoing.

The histological findings support the diagnosis of IgG4-related disease (IgG4-RD). However, according to the 3-step Classification Criteria for IgG4-RD established by the 2019 American College of Rheumatology/European League Against Rheumatism, it would be excluded based solely on its site. This location was intentionally disregarded as it is considered atypical, although the case fulfills the remaining criteria.

## Discussion

Immunoglobulin G4-related disease (IgG4-RD) is an immune-mediated fibroinflammatory disease [1] that can be misdiagnosed as malignancy, infection, or other immune-mediated condition. There are no risk factors neither genetic predilection known.

Involvement has been described in more than 40 different organs from every system in the body [4]. The organs more affected are major salivary glands (submandibular, parotid, sublingual), the orbit and lacrimal glands, the pancreas and biliary tree, the lungs, the kidney, the aorta and the retroperitoneum, the meninges and the thyroid gland. Historically, some manifestations in specific organs were called as Mikulicz’s disease (MD), Kuttner’s tumour, idiopathic orbital inflammation (IOI), multicentric Castleman’s disease-like, Riedel’s thyroiditis and Hashimoto’s thyroiditis (fibrosing subtype).

In 2019, Wallace and colleagues identified four distinct clinical phenotypes of IgG4-RD [6]. Their group was the first to identify that the patients with otherwise different clinical presentations could be segregated into four mutually exclusive, homogeneous groups: Group 1 (pancreatobiliary), group 2 (retroperitoneal fibrosis/aorta), group 3 (head and neck confined), and group 4 (head and neck and systemic).

The head and neck region (H&N) is the second most affected area after the pancreas [7]. We briefly describe the typical head and neck sites affected before focusing on the atypical site manifestations reported in the previous studies.

**Table:** 

**Head and neck typical sites**	
The ACR/EULAR Classification Criteria for IgG4-Related Disease [6] identifies major sites in the head and neck, including salivary glands, orbit, lacrimal glands, thyroid, and meninges, as typical for the condition.	
Salivary glands [8], particularly the submandibular glands [4], are the most frequently affected H&N organs. According to the extension of involvement they were historically divided into two distinct diseases, but nowadays the term “IgG4-related sialadenitis” [9] describes both. The first pattern, named “Mikulicz’s Disease” (MD), typically involves multiple glands, at least two pairs among lacrimal, parotid, submandibular, sublingual and/or minor salivary glands for more than 3 months, usually sparing the parotid gland. The second one, “Kuttner Tumour”, also known as chronic sclerosing sialadenitis, is an isolated firm swelling of one submandibular gland.	
The orbit is the 3rd most involved organ in IgG4-RD after salivary glands and pancreas [4]. In detail, the most involved subsites are the lacrimal glands, usually bilaterally and in association or not with salivary glands as part of MD, followed by the extraocular muscles (EOM), intra or extraconal soft tissue, infraorbital nerve (ION) and eyelids. Historically, orbit involvement by IgG4-RD was called “Sclerosing Subtype Of Idiopathic Orbital Inflammation” or considered part of “Benign Orbital Lymphoproliferative Disorders”. It manifests with increasing periorbital swelling, proptosis, potential diplopia and rarely ophthalmoplegia or impaired visual acuity. Lymphoma is an important differential diagnosis.	
IgG4-related thyroiditis on ultrasound imaging (US) shows heterogeneous echotexture with findings typical of a vascular goiter, which can demonstrate two different patterns: Riedel’s thyroiditis and Hashimoto’s thyroiditis (fibrosing subtype). The former, Riedel’s thyroiditis (RT) also called Riedel-Struma, is a rare disease characterized by chronic inflammation and diffuse fibrosis of the thyroid gland and surrounding tissues. Clinically, it manifests with hypothyroidism and has a “stone-like, hard-as-wood” non-tender consistency upon palpation. It could potentially lead to fibrosclerosis in adjoining organs, such as the the larynx, resulting in obstructive symptom and vocal cold paralysis. The latter, fibrosing Hashimoto’s thyroiditis, is a very rare subtype of Hashimoto’s thyroiditis (HT) where the thyroid gland is enlarged with reduced echogenicity on US, and poorly vascularized. Histopathologically, the thyroid parenchyma shows a higher degree of stromal fibrosis, lymphoplasmacytic infiltrates, and follicular cell degeneration [10]. However, no extrathyroidal extension is seen in contrast to RT.	
Meningeal manifestations include hypertrophic pachymeningitis and intracranial pseudotumor, with differentiation based on extent of inflammatory response [11]. Radiological assessment is crucial, with arteritis suggesting an IgG4-related disease and guiding appropriate management.	
**Head and neck atypical sites**	
The atypical sites of IgG-4 related Disease are less described in literature. We can divide these sites into skull base (excluding meninges, which are a more frequent site), nasal cavities and paranasal sinuses, lymph nodes, and the upper respiratory tract. Virtually every site can be involved.	
**Paranasal sinus**	
Although considered rare, paranasal sinus involvement is the third more common site involved in IgG4D-RD in women [12] while being less frequent in men. It can manifest in two patterns: diffuse rhinosinusitis or local destructive mass-like lesion.	
**IgG4-RD rhinosinusitis**	
When systemic, IgG4-RD can easily be mistaken for common chronic rhinosinusitis (CRS). Moteki et al. [13] demonstrated that the two diseases share a similar immunohistopathological pattern on biopsy. Moreover, symptoms such as nasal obstruction, anosmia, epistaxis, and rhinorrhea, are almost the same. Given these nonspecific characteristics, serum IgG4 level analysis can help in the differentiation diagnosis between them, keeping in mind that IgG4-RD patients are more prone to suffer from sinonasal mucosal disease than the general population.	
**Paranasal mass-like involvement**	
Rather easy to identify, mass-like involvement of IgG4 in paranasal sinuses can be confused with a destructive sinonasal tumor. Maxillary sinuses, followed by ethmoid sinuses, are the most affected [14]. The mass can potentially spread to the adjacent regions, including the skull base, with bone erosion or sclerosis and perineural involvement. Given to this “invasive behaviour”, it is mandatory to require MRI, CT-scan and biopsy to rule out malignancies and invasive fungal sinusitis. Invasive Fungal Sinusitis (IFS) share the same radiological findings [15], that are soft mass-like tissue with or without bone erosion on CT-scan and T1W isointense and T2W hypo-to-isointense on MRI with homogeneous contrast enhancement, but it does not respond to corticosteroids, is related to immunocompromised or diabetic individuals, and has a high mortality rate, up to 80% in the acute form.	
**Oral cavity**	
The oral cavity in rarely affected by IgG4-RD, where it is frequently underrecognized or misdiagnosed. In fact, it is mostly reported in association with other regions, mostly the surrounding salivary glands. Azzi et al. [16] assessed the presence of a total number 51 cases reported in the current literature, although the real number may be underestimated, as many cases were published before ACR/EULAR criteria. The hard palate is the most affected site for oral IgG4-RD [17, 18] and it usually manifests as a long-standing, bilateral rubbery, swelling mass that can be potentially destructive after years [19]. The second site of involvement is the jaw bone [20], which usually presents as an inflammatory pseudotumor with bony destruction. The tongue [21] and the buccal mucosa [22] are both the third most affected subsites that could potentially be misdiagnosed, as a neoplasm or mucositis. Other subsites such as the gingiva, the soft palate, the tonsils, the lips and the base of the tongue alone are less reported in the literature and they can potentially as heterogeneous lesions ranging from erosive and ulcerative masses to granulomas [23]. Radiological findings may include bone resorption, inhomogeneous signal on T2W and irregular contrast enhancement.	
**Lymphonodes**	
Lymphadenopathy is present in up to 70% of IgG4-RD affected patients [24]. In 2012 Y. Sato et al. [25] classified the variable histopathological appearance into five different patterns: Multicentric Castleman’s Disease-Like (Type I) with systemic lymphadenopathy and interfollicular IgG4 plasma cells; reactive follicular hyperplasia-like (type II) with localized lymphadenopathy and interfollicular IgG4 plasma cells; Interfollicular Expansion And Immunoblastosis (Type III) with systemic lymphadenopathy and interfollicular IgG4 plasma cells; Progressively Transformed Germinal Centres-Type (Type IV) with localized or systemic lymphadenopathy and intra-germinal center IgG4 plasma cells, and Inflammatory Pseudotumor-Like (Type V) with localized lymphadenopathy and interfollicular IgG4 plasma cells. Generally, the first-line imaging modality to study infrahyoid lymph nodes is US. On US, IgG4-RD lymphadenopathy usually shows a “pseudo-malignancy” features as rounded shape, loss of central fatty hilum with preserved predominantly central vascularity. However, some subtypes show a pattern of reactive benign lymphadenopathy, with a rather ovoid shape, fatty hilum preservation and central vascular axis as reported by Morisaki et al. [26]. In contrast, CT scan shows aspecific lymphadenopathy/ies: central necrosis isn’t a typical finding. On MRI, involved nodes appear hypointense on T2-weighted imaging and show homogeneous contrast enhancement [27]. FDG-PET shows mild to strong uptake. Obviously, in the presence of lymphadenopathy, the first suspicion should be for malignancy and a differential diagnosis should be made with other conditions such as Rosai-Dorfman disease (RDD) or hyper-interleukin 6 syndromes (including multicentric Castleman disease and rheumatoid arthritis).	
RDD is a rare benign condition consisting into non-clonal histocytes proliferation that could accumulate in lymph nodes and other head and neck sites, whereas hyper-interleukin 6 syndromes, rather called Multicentric Castleman’s disease (MCD) [28] is a lymphoproliferative disorder caused by the hypersecretion of human IL-6 or viral IL-6 by HHV-8 infection (HHV-8 associated MCD) [29], resulting in polyclonal antibody production and plasma cells’ accumulation. Occasionally, MCD can cause serum elevation of IgG4 levels and IgG4-positive plasma cell infiltrates but also lead to systemic symptoms and changes of parameters that could guide to the correct diagnosis. Furthermore, both RDD and MCD have some peculiar histological characteristic that can guide to their recognition.	
**Skull base**	
Skull base involvement in IgG4RD is rare. Some authors have reported cases of temporal bone-middle ear involvement, which clinically and radiologically overlap with otomastoiditis. Patients could complain otorrhea, hearing loss and symptoms of cranial nerve palsy. CT scan shows sclerotic changes in the mastoid cavities, middle ear, and mastoid opacification, with possible bony erosion and invasion of the middle ear structures. It is suggested to carefully exclude infections before exposing patient to immunosuppressive treatment [30].	
In other cases, CT findings reveal a mass-like lesion in the mastoid and middle ear with erosive bony changes. There may also be extension to surrounding structures such as the temporomandibular joint, meninges, jugular foramen carotid canal, pterygopalatine fossa, and nasal cavity.	
Even rarer, an involvement of the cavernous sinus can occur. On MRI, the mass in the cavernous sinus appears hypointense on T2WI, with diffuse enhancement on post contrast T1WI. The mass may exhibit perineural spread and fading into adjacent structures [31].	
**Pituitary gland**	
Also, the pituitary gland can potentially be involved, leading to hypophysitis. Associated pachymeningeal involvement may also be present. Primary hypophysitis is a rare disease and is classified by histologic appearance as lymphocytic, granulomatous, xanthomatous, necrotizing, immunoglobulin G4 (IgG4) plasmacytic, and mixed form. Among these, IgG4-related hypophisitis (IgG4-RH) is the rarest. The first case of IgG4-related hypophysitis diagnosed by pituitary biopsy was reported by Wong in 2007 [32]; however, biopsy in that area is a highly invasive procedure. With the aim of giving a diagnosis avoiding such invasive procedure, in 2011 Leporati [33] created diagnostic criteria for IgG4-RH. In detail, criterion 1 was substantially based on histopathological confirmation; however, if pituitary biopsy was not available, magnetic resonance imaging (MRI) of the pituitary gland (criterion 2) and histopathology of other organs (criterion 3) were sufficient to establish a diagnosis. Simultaneously, if histopathology of other organs was not available, such in cases of isolated IgG4-RH, pituitary MRI (criterion 2), along with increased serum IgG4 level (criterion 4) and prompt response to glucocorticoids (criterion 5) can be used to establish the diagnosis of IgG4-related hypophysitis.	
Regarding pituitary function, in order of prevalence, anterior hypopituitarism, diabetes insipidus and panhypopituitarism may occur. A similar trend is seen on MRI with pituitary enlargement, stalk enlargement and pituitary-stalk enlargement. Yujuan Li et al. [34] reported that anterior hormone deficiency caused by IgG4-RH was, in order of appearance, as follows: gonadotropin > ACTH > TSH > GH, and prolactin, which was different from that of lymphocytic hypophysitis that was ACTH > TSH > gonadotropin > prolactin > GH. Swelling or mass in the pituitary stalk or gland with homogeneous enhancement is often seen on post contrast T1WI.	
**Upper airways**	
Airway involvement has been described in the nasal cavity, trachea, lungs and seldom in the larynx [35]. There are few cases of laryngeal and pharyngeal IgG4-related disease described in the current literature. The most common symptoms are nonspecific, such as dysphonia and dysphagia. Indeed, optic fiber laryngoscopy findings reveal submucosal masses or edematous thickened granular mucosa, that may be localized in different part of the upper airway, leading to symptoms related to mechanical compression. Virk and colleagues previously described a case of subglottic and tracheal stenosis due to a dense fibrous enveloping mass [36].	

Because of these varieties of potential affected sites and lack of reliable specific tests, a diagnosis of IgG4-RD requires a multi-disciplinary collaboration with radiologists and other expert physicians.

Behzadi et al. [37] published a series of institutional 10-years clinical cases and a systematic review of IgGD4 Related Diseases (IgG4-RD) in the head and neck aiming to aid radiologists in diagnosing relapses and new sites of disease. According to their work, IgGD4-RD usually presents on CT scan as an ill-defined mass with homogeneous contrast enhancement. MRI shows low-intermediate T2WI signal and low T1WI signal with homogeneous enhancement. Few cases with isointense to high T2 signal intensity, which correlated with early stages of disease and presence of edematous tissue were reported. Bone erosion and invasion of the nearby structures are also possible findings. Given the fibroinflammatory nature of IgG4 disease, it is not surprisingly FDG avid on PET. Indeed, while US seems not being helpful, a general approach in the imaging workup should include computed tomography or magnetic resonance imaging of the primary site of suspected disease followed, if possible, by biopsy, to sustain the diagnosis and whole-body imaging with CT or FDG-PET/CT to rule out multiorgan disease involvement. Behzadi et al. stress the use of imaging not only for the diagnosis but also in a follow-up setting to evaluate recurrence and/or additional site of disease involvement or possible malignancy onset.

Apart from the current 3-step classification criteria, other authors in the past tried to define the best diagnostic algorithm. For instance, Umehara and Okazaki in 2011 proposed diagnostic criteria based on clinical, biochemical, and histopathological findings [38]. However, the criteria showed to be insufficient because focused on serum level of IgG4, that, as mentioned above, is no longer consider a reliable parameter.

Besides, many efforts have been made to understand the pathophysiology of IgG4-RD as well as to identifay the prognosis factors in aim to offer a target treatment.

Until a few years ago, the pathophysiology of IgG4-RD remained unclear. Nowadays, considerable strides have been made to understand the complexity of these mechanism [39]. Oligoclonal expansion of activated B cells and a cytotoxic subset of CD4 + T cells are the key to the pathological process: IgE and IgG4 plasmoblasts start to secrete self-reactive IgE and IgG4 autoantibodies, circulates in blood flow and partially move to bone marrow as IgE + and IgG4 + resident plasma cells which increase furthermore the serum level of IgE and IgG4. In the affected tissue, expanded CD4 + cytotoxic T lymphocytes (CTLs) and activated macrophages induce apoptotic cell death in cells that bring upregulated MHC class II molecules in the inflammatory environment and secrete peptides and cytokines. Together with the release of PDGF, this led to activation of B cells and, at least, to conversion and promotion of fibroblasts. Fibroblasts secrete extracellular matrix (ECM) that fills the potential space left by apoptotic cell death, leading to fibrosis and tissue remodeling. These remodeling processes explain the typical manifestation of organ enlargement and tumor-like soft tissue masses, whereas histopathological analysis of affected tissue shows lymphoplasmacytic infiltrate and storiform fibrosis.

However, the natural history is controversial. Some involved sites could show spontaneous remission, as seen in AIP (autoimmune pancreatitis) while in other locations, such as submandibular glands (Mikulicz’s diseased) this occurs rarely. It has been demonstrated that overall survival in IgG4D-RD patients is significantly lower than that of the general population, with an estimated odds ratio of 207 [40]. However, the reason is still not completely clarified. We can presume that involvement of vital organ such as heart, kidney and central nervous system leads to significant disfunction. For example, one of the most typical organs involved, the pancreas, may lead to diabetes mellitus. Additionally, diabetes mellitus is strongly correlated with a high cumulative dose of glucocorticoids. Indeed, it is well known the side effects of long-term glucocorticoid therapy: hypertension, osteoporosis, immunosuppression with higher rate of infection, and development of steroid-glucose intolerance.

Carruthers et al. [41] in 2012 proposed an “IgGD4-RD responder index”, a method to quantify the disease activity based essentially on a summary of scores for each affected organ including symptoms, signs, clinical and imaging findings plus a score for serum IgG4 level.

An important issue to consider is the potential association with malignancy. Several studies have reported a threefold risk of solid and hematologic malignancies [42]. However, whether there is an etiological link between IgG4-RD and malignancy is still an open question. In particular, clonal expansion of lymphoid cells in IgG4-RD and/or chronic antigenic stimulation has raised suspicion to be involved in the development of lymphoproliferative disorders, foremost lymphomas [43].

The best treatment for IgG4-RD is currently based on clinical expertise and patient’s condition. Target therapies are not available because the molecular mechanisms underlying the development of IgG4RD still remain unclear.

Also, the lack of randomized controlled trials evaluating the efficacy of different immune-modulatory agents results in the absence of treatment guidelines.

In some asymptomatic cases, a watchful and waiting strategy may be considered. However, cases involving vital organs such as the central nervous system, heard or kidneys may require surgical management to prevent organ damage.

The first-line treatment for active disease is immune-modulating therapy. On the other hand, poor responders are amenable to surgical excision or debulking.

In 2015, forty-two IgG4-RD experts, participated in a study published by the Symposium Organizing Committee on IgG4-Related Disease, which raised the International Consensus Guidance Statement on the Management and Treatment of IgG4-Related Disease [44]. According to their recommendations, glucocorticoids are the first-line agent for inducing remission in every patient with active, untreated IgG4-RD unless contraindications to their use are present. The dosing, timing and tapering regimen varies among studies, but they suggest starting with oral prednisone 0.6 mg/kg per day maintained for 2–4 week and gradually tapering over a period of 3–6 month until remission is achieved. Other experts suggested continuing a low-dose glucocorticoids (5–10 mg/day prednisone or equivalents) up to 3 years [45]. However, as in other immune-mediated disease, relapse rates are quite high after glucocorticoid tapering and glucocorticoids monotherapy may not be sufficient to reach a complete disease control, being long-term toxicities a concern.

A single relapse appears to be a strong predictor of future relapse. To mitigate these side effects, many experts suggest adding conventional steroid-sparing agents (e.g., azathioprine, mycophenolate mofetil, methotrexate, cyclophosphamide) from the beginning of therapy. The same regimen should be used for maintenance therapy. Maintenance therapy is especially beneficial for patients with organ-threatening IgG4-RD manifestations and those bringing an elevated risk of relapse. However, the optimal duration and the best pharmacological strategy require further clarification, as there are currently no randomized controlled trials focused on steroid-conventional agents monotherapy efficacy. Moreover, relapse rates can still occur despite these pharmacological strategies.

At present, IgG4-RD patients with refractory or recurrent disease may be treated with the second-line therapy: rituximab [44]. The use of monoclonal antibodies is increasing due to deeper understanding of IgG4-RD pathophysiology, which has revealed a correlation between plasmoblasts and CD4 + (CD8) cytotoxic T lymphocytes in peripheral blood and tissue and disease activity. Rituximab is monoclonal anti-CD20 antibody that selectively depletes B-cell; it is usually administered intravenously with 100 mg infusion at time zero and at 15th day.

Finally, recent studies have introduced the use of Dupilumab as a steroid-sparing agent. Dupilumab (DUP) is a monoclonal antibody that inhibits interleukin IL-4 and IL-13 signaling and is approved for type 2 Inflammatory Disease such as asthma, chronic rhinosinusitis with nasal polyposis and atopic dermatitis. Kanda et al. [46] reported a case series of four IgG4-RD patient with synchronous type 2 inflammatory disease and IgG4-RD treated with DUP and systemic glucocorticoids (2 patients) and with DUP alone (2 patients). In both groups, serum IgG4 concentration and IgG4-RD responder index decreased considerably, and the former group benefited from glucocorticoids dose reduction. These results could be explained by the role of IL-4 in IgG4 production, inducing IgG4 class-switch mediated by T follicular helper cells and of IL-13, a fibrosis related cytokine that is implicated in IgG4-related sialadenitis.

## Conclusion

IgG4-RD is a multisystemic fibroinflammatory disease that could potentially involve all sites of the body, with a predilection for certain organs such as the major salivary glands, orbits, lacrimal glands, pancreas and biliary tree, kidney, aorta and retroperitoneum, meninges and thyroid gland [4]. Indeed, the head and neck region is frequently affected. Radiologic findings can strongly suggest the correct diagnosis. The 3 step Classification Criteria for IgG4-RD by the 2019 American College of Rheumatology/European League Against Rheumatism [6] is the current flow-chart used to ensure the diagnosis of IgG4-RD in the typical sites of the head and neck. The suspicion of IgG4-RD must be considered in the differential diagnosis when dealing with a lesion in the head and neck region. As evidenced by our case series, the diagnosis of IgG4-RD can be made in many locations, even those less described in the scientific literature and even in locations that international guidelines recognize as atypical. The 3-step Classification Criteria of the guidelines deliberately focus on the most frequent and specific sites for IgG4 disease. The reported cases do not follow the 3-step Classification Criteria, for which they would be excluded solely based on the location. For these reasons, it is of paramount importance that neurosurgeons, head and neck surgeons, and oral and maxillofacial pathologists are familiar with the clinicopathological manifestations of IgG4-RD in these locations to avoid misdiagnosis and inappropriate treatment, which can lead to a decrease in patients’ quality of life.

Fist-line treatment remains glucocorticoids, with rituximab as second-line treatment in refractory and recurrent diseases [44]. However, the optimal duration and the best pharmacological strategy should be clarified. A potential association with malignancy has been reported [47] and is still an open question whether there is an etiologic link between IgG4-RD and the neoplastic process.

As far as we know, there are no risk factors or genetic predispositions. More studies are needed to clarify the pathophysiology of IgG4-RD with the purpose of providing target therapy that could spare steroid-related effects and reduce recurrencies.

## Data Availability

Not applicable.
